# The *Arabidopsis thaliana* proteome harbors undiscovered multi-domain molecules with functional guanylyl cyclase catalytic centers

**DOI:** 10.1186/1478-811X-11-48

**Published:** 2013-07-08

**Authors:** Aloysius Wong, Chris Gehring

**Affiliations:** 1 Division of Biological and Environmental Science and Engineering, 4700 King Abdullah University of Science and Technology, Thuwal 23955-6900, Kingdom of Saudi Arabia

**Keywords:** 3’,5’-cyclic guanosine monophosphate (cGMP), Guanosine-5’-triphosphate (GTP), Guanylyl cyclase (GC), Catalytic center, GC search motif, Homology modeling, Molecular docking

## Abstract

**Background:**

Second messengers link external cues to complex physiological responses. One such messenger, 3’,5’-cyclic guanosine monophosphate (cGMP), has been shown to play a key role in many physiological responses in plants. However, in higher plants, guanylyl cyclases (GCs), enzymes that generate cGMP from guanosine-5’-triphosphate (GTP) have remained elusive until recently. GC search motifs constructed from the alignment of known GCs catalytic centers form vertebrates and lower eukaryotes have led to the identification of a number of plant GCs that have been characterized *in vitro* and *in vivo*.

Presentation of the hypothesis

Recently characterized GCs in *Arabidopsis thaliana* contributed to the development of search parameters that can identify novel candidate GCs in plants. We hypothesize that there are still a substantial number (> 40) of multi-domain molecules with potentially functional GC catalytic centers in plants that remain to be discovered and characterized.

**Testing the hypothesis:**

The hypothesis can be tested, firstly, by computational methods constructing 3D models of selected GC candidates using available crystal structures as templates. Homology modeling must include substrate docking that can provide support for the structural feasibility of the GC catalytic centers in those candidates. Secondly, recombinant peptides containing the GC domain need to be tested in *in vitro* GC assays such as the enzyme-linked immune-sorbent assay (ELISA) and/or in mass spectrometry based cGMP assays. In addition, quantification of *in vivo* cGMP transients with fluorescent cGMP-reporter assays in wild-type or selected mutants will help to elucidate the biological role of novel GCs.

Implications of the hypothesis

If it turns out that plants do harbor a large number of functional GC domains as part of multi-domain enzymes, then major new insights will be gained into the complex signal transduction pathways that link cGMP to fundamental processes such as ion transport and homeostasis, biotic and abiotic stress responses as well as cGMP-dependent responses to hormones.

## Background

While cGMP is increasingly accepted as an important signaling component in many plant responses e.g. [1-3], it is perhaps astonishing that the discovery and functional characterization of GCs in higher plants is only just beginning, particularly so since in single celled green alga *Chlamydomonas reinhardtii* there are > 90 annotated nucleotide cyclases (NCs) that come in > 20 different domain combinations with 13 different domain partners [4]. The structural diversity and complexity of molecules with NC activity [4-6] are one likely reason why BLAST searches with known NCs from lower and higher eukaryotes did not yield candidate molecules in higher plants.

Search strategies based on conserved and functionally assigned amino acid (AA) residues in the catalytic center of known NCs [7] have now opened the way to a systematic search of NCs in higher plants and has led to the discovery of a number of *Arabidopsis thaliana* candidate molecules with catalytic activity *in vitro* and *in vivo*. These molecules include a wall-associated kinase like protein (AtWAKL10) with a role in defense [8], the brassinosteroid receptor (AtBRI1) [9], the Pep1 receptor (AtPepR1) [10] and the phytosulfokine receptor (AtPSKR) [11] as well as a nitric oxide-binding GC (AtNOGC1) [12]. PSKR belongs to a family of NCs that contains the GC catalytic center embedded within the intracellular kinase domain of leucine rich repeat receptor-like molecules and in *in vitro* experiments we have demonstrated that both the kinase and the GC domain have catalytic activity. Importantly, the natural ligands for both the PSKR and BRI1 receptors increase intracellular cGMP levels in isolated mesophyll protoplast assays suggesting that the GC activity is functionally relevant *in planta*[6,11].

### Presentation of the hypothesis

**Figure 1:**
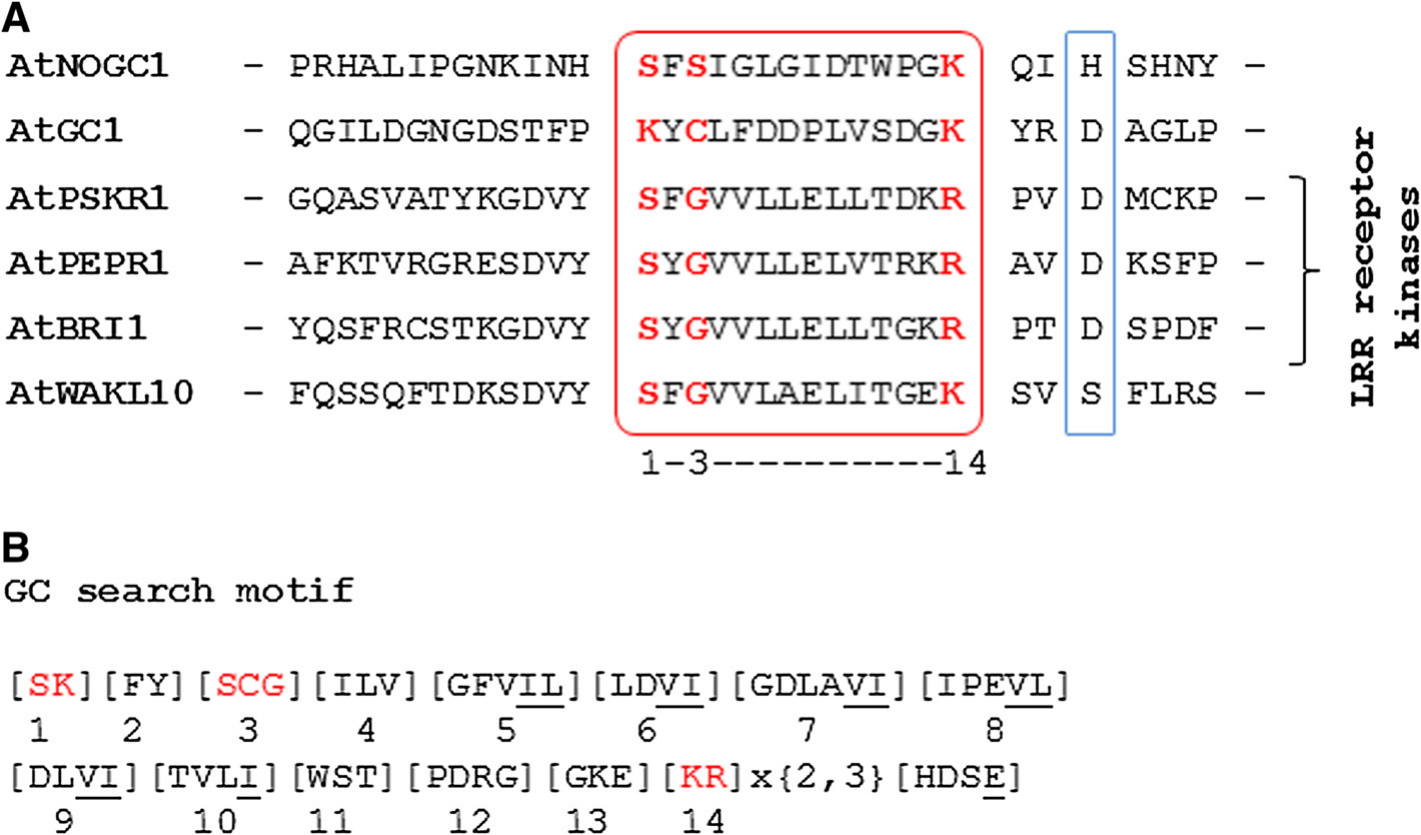
**Construction of a GC search motif based on the catalytic center of characterized GCs in *Arabidopsis thaliana*.** The GC catalytic centers in the alignment of all previously characterized GCs in Arabidopsis **(A)**, is represented in the red box while the blue box implicates binding with Mg^2+^ or Mn^2+^ ions. A 14 amino acid long GC search motif (**B**) was built by including all the residues present in each position of the catalytic center. The amino acid substitutions are in square brackets ([ ]), “X” stands for any amino acid and the gap size is marked in curly brackets ({ }). Underlined amino acids have been added to the motif because of their chemical similarity to the amino acid at this position. Amino acids highlighted in red are functionally assigned residues; amino acid in position 1 forms the hydrogen bond with the guanine, amino acid in position 3 confers substrate specificity for GTP while amino acid in position 14 stabilizes the transition state from GTP to cGMP. The identification and assignment of functions to key residues of the GC motif are detailed in [7].

We propose that in addition to the six characterized GCs in *Arabidopsis thaliana* to-date (AtGC1, AtNOGC1, AtPSKR1, AtPEPR1, AtBRI1 and AtWAKL10), higher plants harbor a substantially larger number of GCs that remain to be discovered. This hypothesis is based on the fact that the tested GCs share a distinct AA signature in the catalytic center (Figure 1A) where the AA at position 1 forms the hydrogen bond with the guanine, the residue in position 3 confers substrate specificity for GTP while the AA in position 14 stabilizes the transition state from GTP to cGMP and two or three AAs away from the C-terminal end of the motif is the residue that interacts with the Mg^2+^/Mn^2+^ ions (Figure 1B). This motif based on tested GCs (Figure 1B) identifies 41 novel Arabidopsis candidate GCs (see Additional file 1) (http://www.arabidopsis.org/cgi-bin/patmatch/nph-patmatch.pl) [13] and no proteins in kingdoms other than the Viridiplantae. However, we do not predict that all retrieved candidate GCs will have activity *in vitro* and/or *in vivo*, nor do we exclude that molecules that do not contain the motif function as GCs.

### Testing the hypothesis

**Figure 2:**
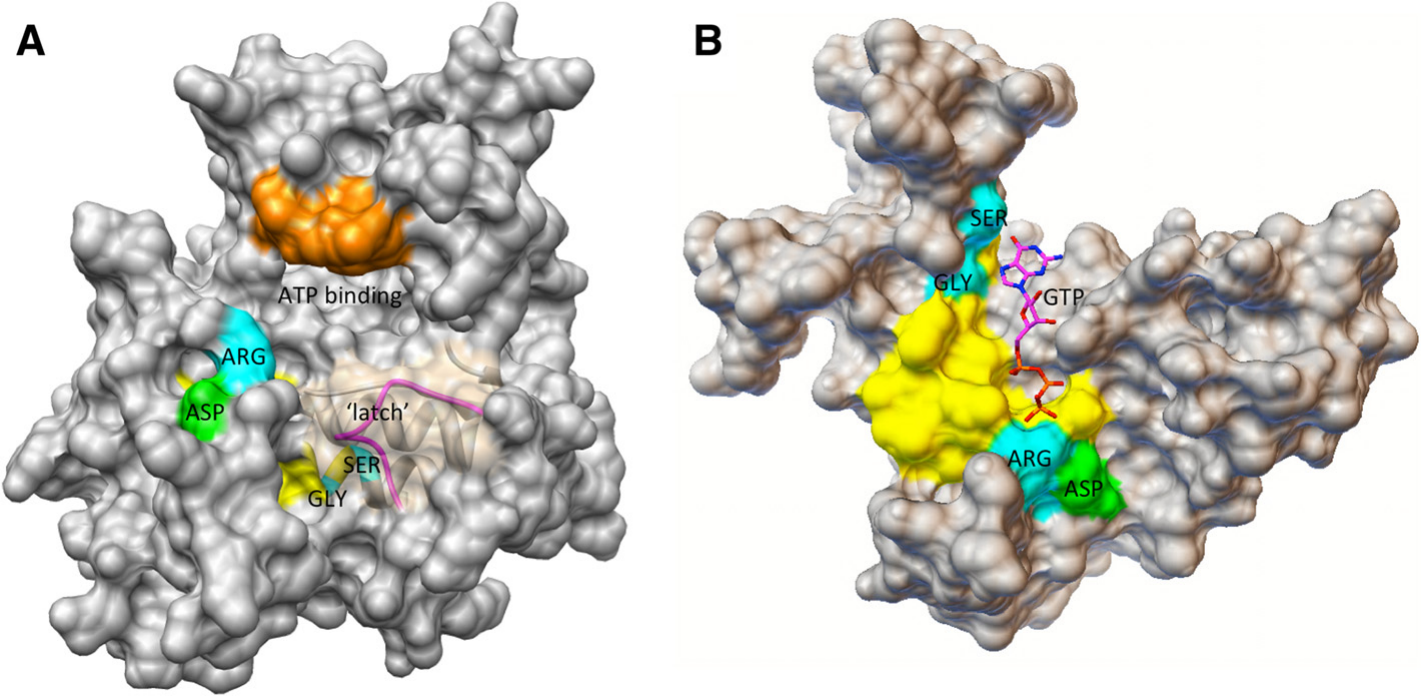
**Computational assessment of the predicted activation states of AtPSKR1 kinase and GC catalytic centers.** AtPSKR1-kinase domain (Phe^734^ – Val^1008^) was modeled against the AvrPtoB-BAK1 complex (PDB entry: 3TL8) **(A)** and against the bacteria GC Cya2 (PDB entry: 2W01) **(B)** using the Modeller (ver. 9.10) software [14], and GTP docking experiments performed using AutoDock Vina (ver. 1.1.2) [15]. When AtPSKR1 assumes the kinase activation state **(A)**, the GC catalytic center is partially covered by a nine amino acid long ‘latch’ that may be loosened by a molecular switch (e.g. Ca^2+^) or by forming homo- or hetero-dimers. When AtPSKR1 assumes a GC configuration **(B)**, the GC catalytic center is completely exposed and assessable to GTP. In addition, automated docking experiments suggest feasible GTP docking. The GC catalytic center is in yellow, functionally assigned residues in the GC motif are in cyan, amino acids implicated in metal binding are in green, the ATP-binding site is in orange and the nine amino acid long ‘latch’ partially covering the GC catalytic center is in magenta.

**Figure 3:**
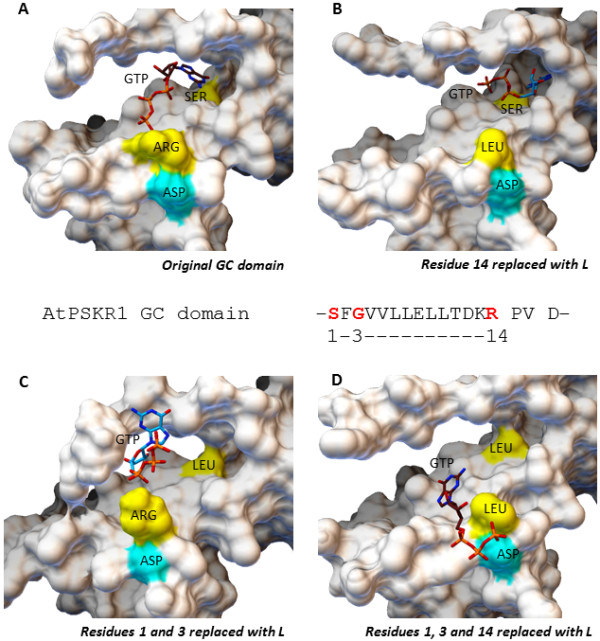
**Assessment of the AtPSKR1 GC catalytic center by molecular docking of GTP.** According to the 3D model of AtPSKR1, the GC domain is embedded within a cavity with the key residues for GTP interaction and the metal binding residue highlighted in yellow and cyan respectively. The GTP substrate docks successfully with the guanine portion at the inner-most of the cavity for interaction with the serine residue (position 1) and the phosphate end pointing outwards of the cavity towards the arginine residue (position 14) - an orientation deemed suitable for GC activity **(A)**. When one or more key amino acid residues at position 1, 3 and/or 14 was replaced with leucine, docking experiments indicate disruptions in the docking orientations of GTP in all except for the leucine replacements at positions 1 and 3 of the GC domain. This suggests an aberrant GC catalytic activity when these key residues are replaced. GTP docking results for AtPSKR1 GC domain with leucine replacements at position 14 **(B)**, positions 1 and 3 **(C)**, and positions 1, 3 and 14 **(D)** are as illustrated. AtPSKR1-GCD (Asn^871^ – Glu^980^) was modeled against the AvrPtoB-BAK1 complex (PDB entry: 3TL8) using the Modeller (ver. 9.10) software [14] while GTP docking experiments were performed using AutoDock Vina (ver. 1.1.2) [15]. The full-length AtPSKR1 protein and the domains organization are as shown in Additional file 2.

Firstly, the hypothesis can be tested by using computational approaches to predict the structural properties of candidate GCs and testing should include automated substrate (GTP) docking protocols. To obtain insights on the structural features of the GC catalytic centers, 3D models can be constructed using readily available crystal structures as templates. A high sequence similarity between subject and template will generate accurate 3D models. While sequence similarity of 25% is sufficient for structural predictions, we recommend selecting templates with a BLAST alignment score of at least 50–80 (color key: green). For example, a 3D structure of the GC region of AtPSKR1 built using homology modeling techniques revealed that the GC catalytic center is embedded within a cavity, presumably providing ideal steric interactions for substrate docking. While selecting template structures with the highest sequence similarity to the candidate GCs is the common practice, we would recommend to also evaluate the candidate proteins against a known GC template (e.g. crystal structure of a bacterial or human GC) since many plant candidate GCs are embedded within kinases, and modeling against a kinase template may not reflect the configuration of an activated candidate GC. The reason is that dual-activity enzymes such as AtPSKR1 do not assume concurrent activation states for both the kinase and GC catalytic domains, and it is likely that a molecular switch (e.g. Ca^2+^ and/or dimerization with another molecule) is required to shift from kinase to GC activation. In the example here, when the AtPSKR1 molecule is in the kinase state the GC catalytic center is partially buried. In turn, when the molecule is in the GC configuration, the GC catalytic center is completely exposed and GTP can successfully dock (as predicted by AutoDock Vina) (Figure 2). GTP docking at the PSKR1 GC center in the correct orientation favorable for interactions with the key residues within the cavity is represented in Additional file 2: Figure 3A. Functionally assigned residues of the GC motif can be replaced with another amino acid to estimate the importance or relevance of these residues in maintaining a functional configuration of the catalytic center. For example, when one or more key residues in the AtPSKR1 GC domain is replaced with leucine, GTP is more likely to fail to dock or dock in the wrong orientation, implying compromised or abolished GC activity (Figure 3B-D). In previously characterized Arabidopsis GCs, GTP docking is also disrupted when key residues are replaced (Table 1). We note that these computational methods alone are not diagnostic of GC activity and do not distinguish GCs from other enzymes that also catalyze GTP (e.g. GTPases). They however lend good support to the experimental data and can be used as an initial screen to assist in the selection of candidate molecules from a potentially large pool of proteins for subsequent *in vitro* and/or *in vivo* enzymatic functional assays.

**Table 1:** Molecular docking of GTP with the GC catalytic center

	**Amino acid position in the GC motif replaced with leucine (L)**						
	**1**	**3**	**14**	**1,3**	**1,14**	**3,14**	**1,3,14**
AtPSKR1	✓	✓	✗	✗	✗	✗	✗
AtPEPR1	✗	✗	✗	✗	✗	✗	✗
AtBRI1	✓	✗	✗	✗	✓	✗	✗
AtWAKL10	✗	✗	✗	✗	✗	✗	✗

Functionally-important amino acid residues at position 1, 3 and/or 14 of the GC motif were replaced with leucine for homology modeling and docking experiments.

✓ indicates successful docking of GTP and in an orientation deemed suitable for catalysis.

✗ indicates unsuccessful docking of GTP or GTP docking in an orientation deemed unsuitable for catalysis.

Secondly, the candidate GCs need to be tested *in vitro* by incubating the recombinant protein harboring the GC domain with GTP and the appropriate metal ions (Mg^2+^ and/or Mn^2+^). This recombinant protein can be made by molecular cloning methods with the DNA construct expressed in an *E. coli* host system and affinity purified for the following *in vitro* GC activity testing. GC enzymatic reaction is initiated by incubating the purified recombinant protein in buffer containing the aforementioned ingredients. cGMP generation can then be measured using commercially available cGMP immunoassay kits which will provide an indication of GC activity. Cyclic GMP production should be further verified using mass spectrometry based techniques that consistently record higher cGMP amounts in independent *in vitro* experiments than those obtained with ELISA-based assays [9]. The substantially lower *in vitro* activities of plant GCs compared to animal GCs [5] have raised concerns regarding (1) the folding and structural integrity of the recombinant plant GCs and (2) the reliability of the *in vitro* recombinant GC activity assays to detect such low amounts of cGMP [16,17]. We therefore recommend that candidate GCs be evaluated with both the biochemical assays and the more sensitive mass spectrometric methods.

Thirdly, candidate GC candidates should also be studied *in vivo*[18] with fluorescent cGMP-reporter assays, since this is a direct way to link ligand–binding to receptor-coupled GCs, and to the generation of cGMP and cGMP-dependent downstream effects.

### Implications of the hypothesis

Currently, we know that a growing number of fundamental physiological processes, including gating of ion channels [19], specific phosphorylation events [18], post-translational modifications [20], stomatal guard cell movements and responses to hormones [18,21] all depending, at least in part, on cGMP. Consequently, a major outstanding question is, where are the enzymes that catalyze the reaction from GTP to cGMP, how many are there in e.g. Arabidopsis, and how are they regulated? If our hypothesis proves right, and novel multi-domain enzymes with stimulus- and/or ligand-specific GC activity will be discovered, we will be able to finally unravel the complex signal transduction networks that link environmental stimuli to cGMP-dependent responses in plants such as chloroplast development and anthocyanin synthesis [22,23]. Detailed analysis of cGMP-dependent responses will have to include a genetic and molecular analysis of transgenic plants with overexpressing or knocked-down of candidate GCs as well as transcriptomics studies that reveal further aspects and the extent to which cGMP modulates down-stream effects. In addition, and given the importance of cGMP-dependent phosphorylation, we would argue that comparative phospho-proteomics of wild type and GC mutants will provide a systems view of specific phosphorylation cascades that are induced by the activation of target GCs. Taken together, we predict, that if our hypothesis is true and the candidate GCs will be analyzed in considerable depth, it will establish cGMP, much like cytosolic free Ca^2+^, as a key second messenger in plant responses.

## Abbreviations

cGMP: 3’,5’-cyclic guanosine monophosphate; GTP: Guanosine-5’-triphosphate; GC: Guanylyl cyclase; BLAST: Basic local alignment search tool; ELISA: Enzyme-linked immune-sorbent assay.

## Competing interests

The authors declare that they have no competing interests.

## Authors’ contributions

CG conceived the project. AW performed the modeling and bioinformatics analysis, and CG and AW wrote the manuscript. All authors read and approved the final manuscript.

## Supplementary Material

Additional file 1 The file contains the pattern matching (patmatch) search parameters and a list of the 41 new candidates retrieved using the proposed GC motif. Click here for file

Additional file 2 The file contains a model of the full-length AtPSKR1 with the respective LRR, kinase and GC domains represented. Click here for file

## References

[B1] Donaldson L Ludidi N Knight MR Gehring C Denby K Salt and osmotic stress cause rapid increases in Arabidopsis thaliana cGMP levels FEBS Lett 2004 569 317 320 10.1016/j.febslet.2004.06.016 15225654

[B2] Maathuis FJ cGMP modulates gene transcription and cation transport in Arabidopsis roots Plant J 2006 45 700 711 10.1111/j.1365-313X.2005.02616.x 16460505

[B3] Pasqualini S Meier S Gehring C Madeo L Fornaciari M Romano B Ederli L Ozone and nitric oxide induce cGMP-dependent and -independent transcription of defence genes in tobacco New Phytol 2009 181 860 870 10.1111/j.1469-8137.2008.02711.x 19140946

[B4] Meier S Seoighe C Kwezi L Irving H Gehring C Plant nucleotide cyclases: an increasingly complex and growing family Plant Signal Behav 2007 2 536 539 10.4161/psb.2.6.4788 19704552 PMC2634362

[B5] Gehring C Adenyl cyclases and cAMP in plant signaling - past and present Cell Commun Signal 2010 8 15 10.1186/1478-811X-8-15 20579354 PMC2907374

[B6] Irving HR Kwezi L Wheeler J Gehring C Moonlighting kinases with guanylate cyclase activity can tune regulatory signal networks Plant Signal Behav 2012 7 201 204 10.4161/psb.18891 22353864 PMC3405710

[B7] Ludidi N Gehring C Identification of a novel protein with guanylyl cyclase activity in Arabidopsis thaliana J Biol Chem 2003 278 6490 6494 10.1074/jbc.M210983200 12482758

[B8] Meier S Ruzvidzo O Morse M Donaldson L Kwezi L Gehring C The Arabidopsis wall associated kinase-like 10 gene encodes a functional guanylyl cyclase and is co-expressed with pathogen defense related genes PLoS One 2010 5 e8904 10.1371/journal.pone.0008904 20126659 PMC2811198

[B9] Kwezi L Meier S Mungur L Ruzvidzo O Irving H Gehring C The Arabidopsis thaliana brassinosteroid receptor (AtBRI1) contains a domain that functions as a guanylyl cyclase in vitro PLoS One 2007 2 e449 10.1371/journal.pone.0000449 17520012 PMC1867859

[B10] Qi Z Verma R Gehring C Yamaguchi Y Zhao Y Ryan CA Berkowitz GA Ca^2+^ signaling by plant Arabidopsis thaliana Pep peptides depends on AtPepR1, a receptor with guanylyl cyclase activity, and cGMP-activated Ca^2+^ channels Proc Natl Acad Sci U S A 2010 107 21193 21198 10.1073/pnas.1000191107 21088220 PMC3000296

[B11] Kwezi L Ruzvidzo O Wheeler JI Govender K Iacuone S Thompson PE Gehring C Irving HR The phytosulfokine (PSK) receptor is capable of guanylate cyclase activity and enabling cyclic GMP-dependent signaling in plants J Biol Chem 2011 286 22580 22588 10.1074/jbc.M110.168823 21504901 PMC3121402

[B12] Mulaudzi T Ludidi N Ruzvidzo O Morse M Hendricks N Iwuoha E Gehring C Identification of a novel Arabidopsis thaliana nitric oxide-binding molecule with guanylate cyclase activity in vitro FEBS Lett 2011 585 2693 2697 10.1016/j.febslet.2011.07.023 21820435

[B13] Yan T Yoo D Berardini TZ Mueller LA Weems DC Weng S Cherry JM Rhee SY PatMatch: a program for finding patterns in peptide and nucleotide sequences Nucleic Acids Res 2005 33 W262 W266 10.1093/nar/gki368 15980466 PMC1160129

[B14] Sali A Blundell TL Comparative protein modelling by satisfaction of spatial restraints J Mol Biol 1993 234 779 815 10.1006/jmbi.1993.1626 8254673

[B15] Trott O Olson AJ AutoDock Vina: improving the speed and accuracy of docking with a new scoring function, efficient optimization, and multithreading J Comput Chem 2010 31 455 461 19499576 10.1002/jcc.21334 PMC3041641

[B16] Ashton AR Guanylyl cyclase activity in plants? Proc Natl Acad Sci U S A 2011 108 E96 author reply E97-98 10.1073/pnas.1101007108 21527716 PMC3093516

[B17] Berkowitz GA Gehring C Irving HR Kwezi L Reply to Ashton: The putative guanylyl cyclase domain of AtPepR1 and similar plant receptors Proc Natl Acad Sci U S A 2011 108 E97 E98 10.1073/pnas.1103313108

[B18] Isner JC Nuhse T Maathuis FJ The cyclic nucleotide cGMP is involved in plant hormone signalling and alters phosphorylation of Arabidopsis thaliana root proteins J Exp Bot 2012 63 3199 3205 10.1093/jxb/ers045 22345640 PMC3350932

[B19] Zelman AK Dawe A Gehring C Berkowitz GA Evolutionary and structural perspectives of plant cyclic nucleotide-gated cation channels Front Plant Sci 2012 3 95 22661976 10.3389/fpls.2012.00095 PMC3362136

[B20] Marondedze C Turek I Parrott B Thomas L Jankovic B Lilley KS Gehring C Structural and functional characteristics of cGMP-dependent methionine oxidation in Arabidopsis thaliana proteins Cell Commun Signal 2013 11 1 10.1186/1478-811X-11-1 23289948 PMC3544604

[B21] Pharmawati M Billington T Gehring CA Stomatal guard cell responses to kinetin and natriuretic peptides are cGMP-dependent Cell Mol Life Sci 1998 54 272 276 10.1007/s000180050149 9575339 PMC11147171

[B22] Bowler C Yamagata H Neuhaus G Chua NH Phytochrome signal transduction pathways are regulated by reciprocal control mechanisms Genes Dev 1994 8 2188 2202 10.1101/gad.8.18.2188 7958888

[B23] Bowler C Neuhaus G Yamagata H Chua NH Cyclic GMP and calcium mediate phytochrome phototransduction Cell 1994 77 73 81 10.1016/0092-8674(94)90236-4 8156599

