# Supplementary material for: The Arabidopsis thaliana proteome harbors undiscovered multi-domain molecules with functional guanylyl cyclase catalytic centers
Source: Cell Commun Signal. 2013 Jul 8;11:48. doi: 10.1186/1478-811X-11-48 (PMC3726316; doi:10.1186/1478-811X-11-48)
Supplement: Additional file 1 — The file contains the pattern matching (patmatch) search parameters and a list of the 41 new candidates retrieved using the proposed GC motif. [file 1478-811X-11-48-S1.pdf]

ADDITIONAL FILE 1

Pattern Matching (PatMatch) search parameters:

Search pattern	[KS][YF][GCS][VIL][VILFG][DVIL][VILADG][EPVIL][DVIL][TVIL][WST][PDRG][KEG][KR]x{2,3}[DHSE]
Dataset searched	TAIR10 Proteins (protein)
Bytes searched	14482855
Sequences with hits	47
Hits found	47

Retrieved hits:

AT1G16110.1	(AtWAKL6)	620	636	SFGVVLVELITGEKPLS
AT1G16120.1	(AtWAKL1)	615	631	SFGVVLVELITGEKPLS
AT1G16130.1	(AtWAKL2)	602	618	SFGVVLVELLTGEKPSS
AT1G16160.1	(AtWAKL5)	598	614	SFGVVLVELITGEKPLS
AT1G16260.1	(AtWAKL)	576	592	SFGVLLIELLTGEKPVS
AT1G16260.2	(AtWAKL)	576	592	SFGVLLIELLTGEKPVS
AT1G17750.1	(AtPEPR2)	981	997	SYGVVLLELVTGKRALD
AT1G28440.1	(AtHSL1)	878	894	SFGVVILEIVTRKRPVD
AT1G31420.1	(AtFEI1)	491	507	SFGVLVLEVLSGKRPTD
AT1G31420.2	(AtFEI1)	490	506	SFGVLVLEVLSGKRPTD
AT1G52540.1	(Kinase)	226	242	SFGVLLLELVTGKRPTE
AT1G55610.1	(AtBRI1-L)	1049	1065	SYGVILLELLSGKKPID
AT1G55610.2	(AtBRI1-L)	1049	1065	SYGVILLELLSGKKPID
AT1G61430.1	(Kinase-L)	679	695	SFGVLLLEIISGKKISS
**AT1G62580.1**	**(AtNOGC1)**	**191**	**207**	**SFSIGLGIDTWPGKQIH**
AT1G69270.1	(AtRPK1)	446	462	SYGIVLLELISDKRALD
AT1G69730.1	(AtWAKL)	634	650	SFGVVLVELITGEKSIS
**AT1G73080.1**	**(AtPEPR1)**	**1014**	**1030**	**SYGVVLLELVTRKRAVD**
AT1G79670.1	(AtWAKL22)	607	623	SFGVVLVELITGEKPFS
AT1G79670.2	(AtWAKL22)	570	586	SFGVVLVELITGEKPFS
**AT1G79680.1**	**(AtWAKL10)**	**620**	**636**	**SFGVVLAELITGEKSVS**
**AT2G02220.1**	**(AtPSKR1)**	**921**	**937**	**SFGVVLLELLTDKRPVD**
AT2G26290.1	(AtARSK1)	280	296	SFGVVLLELITGKRSMD
AT2G39660.1	(AtBIK1)	264	280	SFGVLLLEILSGKRALD
AT3G02130.1	(AtCLI1)	1057	1073	SYGVVLLELLSDKKALD
AT3G13380.1	(AtBRI1-L)	1047	1063	SYGVILLELLSGKKPID
AT3G19300.1	(Kinase-L)	514	530	SYGVVLLEIITGKRAVD
AT3G49670.1	(AtBAM2)	878	895	SFGVVLLELITGKKPVGE
AT4G05200.1	(AtCRK22)	534	550	SFGVLVLELITGKKNSS
AT4G11530.1	(AtCRK34)	532	548	SFGVLVLEIISGKKNSS
AT4G13190.1	(Kinase-L)	260	276	SFGVVLLELITGKRVID
AT4G21390.1	(AtB120)	716	732	SFGVLLLEIVSGKRNTS
AT4G23180.1	(AtCRK10)	535	551	SFGVLVLEIISGKKNSS
AT4G23230.1	(AtCRK15)	404	420	SFGVLVLEIISGKKNNS
AT4G23250.1	(AtCRK17)	541	557	SFGVLILEIISGKKNSS
AT4G23260.1	(AtCRK18)	526	542	SFGVLILEIISGKKNSS
AT4G23260.2	(AtCRK18)	515	531	SFGVLILEIISGKKNSS
AT4G23300.1	(AtCRK22)	540	556	SFGILILEIISGKKNSS
**AT4G39400.1**	**(AtBRI1)**	**1071**	**1087**	**SYGVVLLELLTGKRPTD**
**AT5G05930.1**	**(AtGC1)**	**32**	**48**	**KYCLFDDPLVSDGKYRD**
AT5G07180.1	(AtERL2)	837	853	SFGIVLLELLTGKKAVD
AT5G58150.1	(Kinase-L)	702	719	SFGVVLLELVSGKKPEGD
AT5G61480.1	(AtPXY)	911	927	SYGVILLEIITGKRSVE
AT5G62230.1	(AtERL1)	834	850	SFGIVLLELLTGKKAVD
AT5G62230.2	(AtERL1)	786	802	SFGIVLLELLTGKKAVD
AT5G62710.1	(Kinase-L)	499	515	SFGVLLLELVTGKRPTD
AT5G65710.1	(AtHSL2)	883	899	SFGVVLLELITGKRPND

Legend:
Bolded proteins – Previously reported GCs

Note:
Leucine-rich repeats (LRR), cysteine-rich kinases (CRK) and wall-associated kinase-like (WAKL) proteins constitute 20, 8 and 9 of the new candidate GCs respectively.
