# Supplementary material for: The Arabidopsis thaliana proteome harbors undiscovered multi-domain molecules with functional guanylyl cyclase catalytic centers
Source: Cell Commun Signal. 2013 Jul 8;11:48. doi: 10.1186/1478-811X-11-48 (PMC3726316; doi:10.1186/1478-811X-11-48)
Supplement: Additional file 2 — The file contains a model of the full-length AtPSKR1 with the respective LRR, kinase and GC domains represented. [file 1478-811X-11-48-S2.pdf]

ADDITIONAL FILE 2

Full-length AtPSKR1 structure modeled against the BRI1 ectodomain (PDB ID: 3RIZ) and the organization of the multiple domains

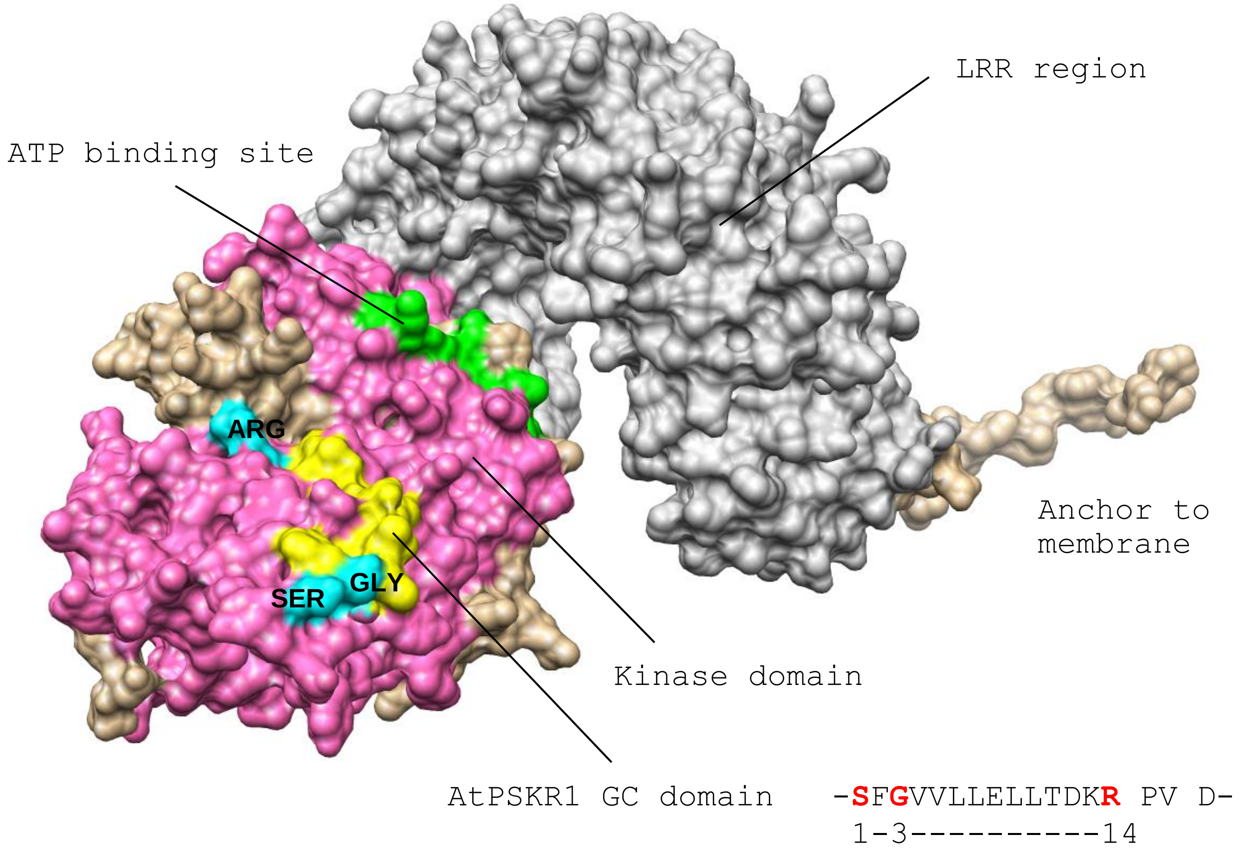

Legend:
Grey LRR region
Pink Kinase domain
Yellow GC catalytic center
Green ATP binding site
Cyan Functionally-assigned key residues in the GC motif

Note:
This full-length PSKR1 model only serves to depict the domain organization of the multiple domains and to check for possible structural interference which may hinder substrate docking at the catalytic center. For an accurate model of the PSKR1-GC catalytic center, please refer to Figure 2. Both the ATP binding site and the GC catalytic center seem to be sufficiently exposed for substrate interactions.
